# Definitions of poor outcome after total knee arthroplasty: an inventory review

**DOI:** 10.1186/s12891-020-03406-y

**Published:** 2020-06-13

**Authors:** Malou E. M. te Molder, José M. H. Smolders, Petra J. C. Heesterbeek, Cornelia H. M. van den Ende

**Affiliations:** 1 Sint Maartenskliniek Research, Sint Maartenskliniek, P.O. box 9011, 6500 GM Nijmegen, The Netherlands; 2 Department of Orthopedics, Radboud University Medical Center, Nijmegen, The Netherlands; 3 Department of Orthopedic Surgery, Sint Maartenskliniek, Nijmegen, The Netherlands; 4 Department of Rheumatology, Sint Maartenskliniek, Nijmegen, The Netherlands; 5 Department of Rheumatology, Radboud University Medical Center, Nijmegen, The Netherlands

**Keywords:** Total knee arthroplasty, Treatment outcome, Poor outcome, Patient-reported outcome measures, Osteoarthritis knee

## Abstract

**Background:**

A significant proportion of patients experiences poor response (i.e. no or little improvement) after total knee arthroplasty (TKA) because of osteoarthritis. It is difficult to quantify the proportion of patients who experiences poor response to TKA, as different definitions of, and perspectives (clinician’s and patient’s) on poor response are being used. The aim of this study was therefore to review the literature and summarize definitions of poor response to TKA.

**Methods:**

A systematic search was performed to identify and review studies that included dichotomous definitions of poor outcome after primary TKA. The type, amount and combination of domains (e.g. functioning), outcome measures, type of thresholds (absolute/relative, change/cut-off), values and moments of follow-up used in definitions were summarized.

**Results:**

A total of 47 different dichotomous definitions of poor response to TKA were extracted from 2163 initially identified studies. Thirty-six definitions incorporated one domain, seven definitions comprised two domains and four definitions comprised three domains. Eight different domains were used in identified definitions: pain, function, physical functioning, quality of life (QoL), patient satisfaction, anxiety, depression and patient global assessment. The absolute cut-off value was the most common type of threshold, with large variety in value and timing of follow-up.

**Conclusions:**

Our inventory review shows that definitions of poor response to TKA are heterogeneous. Our findings stresses the need for an unambiguous definition of poor response to draw conclusions about the prevalence of poor-responders to TKA across hospitals and countries, and to identify patients at risk.

## Background

Total knee arthroplasty (TKA) is considered to be a cost-effective intervention for the treatment of advanced knee osteoarthritis (OA). Nonetheless, increasing evidence shows that a significant proportion of patients experiences a poor response to TKA (i.e. show no or little improvement) in terms of chronic knee pain [1, 2], functional disability [3, 4], poor quality of life (QoL) [5], and dissatisfaction after TKA [6–13]. However, both in research and in clinical practice it is challenging to identify those patients with an unfavourable course after TKA, as different definitions of non-response are being used.

The comparison of research findings on the effectiveness of TKA across studies and countries is hampered by the fact that different concepts for (poor) response for outcome after TKA are being used [14]. Various concepts or definitions of (poor) response are developed on group level, using mean changes, to describe improvement over time in patient cohorts. Furthermore, there might be differences in perspective of concepts of (poor) response among different stakeholders (i.e. physician, patient, clinical researcher or health insurer). Physicians usually focus on aspects of a dimension based on their clinical evaluation (e.g. stability, range of motion and alignment), while patients focus on the functionality of the knee during daily life activity [12]. Moreover, the view of physicians and patients on the desired magnitude of improvement after TKA are not always consistent [13], as poor correlations were found between physician-assessed and patient-reported outcome measures (PROMs) [15]. Research findings on the outcome of TKA are predominantly based on single continuous outcome measures assessing one construct, without taking all potential relevant constructs into account. A preliminary set of domains for total joint replacement (TJR) clinical trials was proposed by international experts that included pain, function, patient satisfaction, revision, adverse events, and death [16], but no propositions were made to what extent these domains should be incorporated in a definition of (poor) response to TKA.

Currently, it is unclear which definitions of poor response to primary TKA are used in the literature. This insight can help to reach consensus on an unambiguous definition of poor response. The need to use a combined endpoint incorporating relevant constructs, and a relevant amount of change based on multiple, clinical outcome perspectives (including physicians’ and patients’ outcome perspective) to accurately describe poor-response to TKA [17], has been recognized in the literature.

The aim of this study was to review and summarize dichotomous definitions of poor response as dichotomous definitions allow to make inferences about the prevalence of poor outcome and comparisons of TKR outcome across hospitals, countries and over time.

We expect a variety of definitions and outcomes used to define poor response after primary TKA. Therefore this study systematically map definitions of poor response to primary TKA in the literature.

## Methods

### Search strategy

**Fig. 1:**
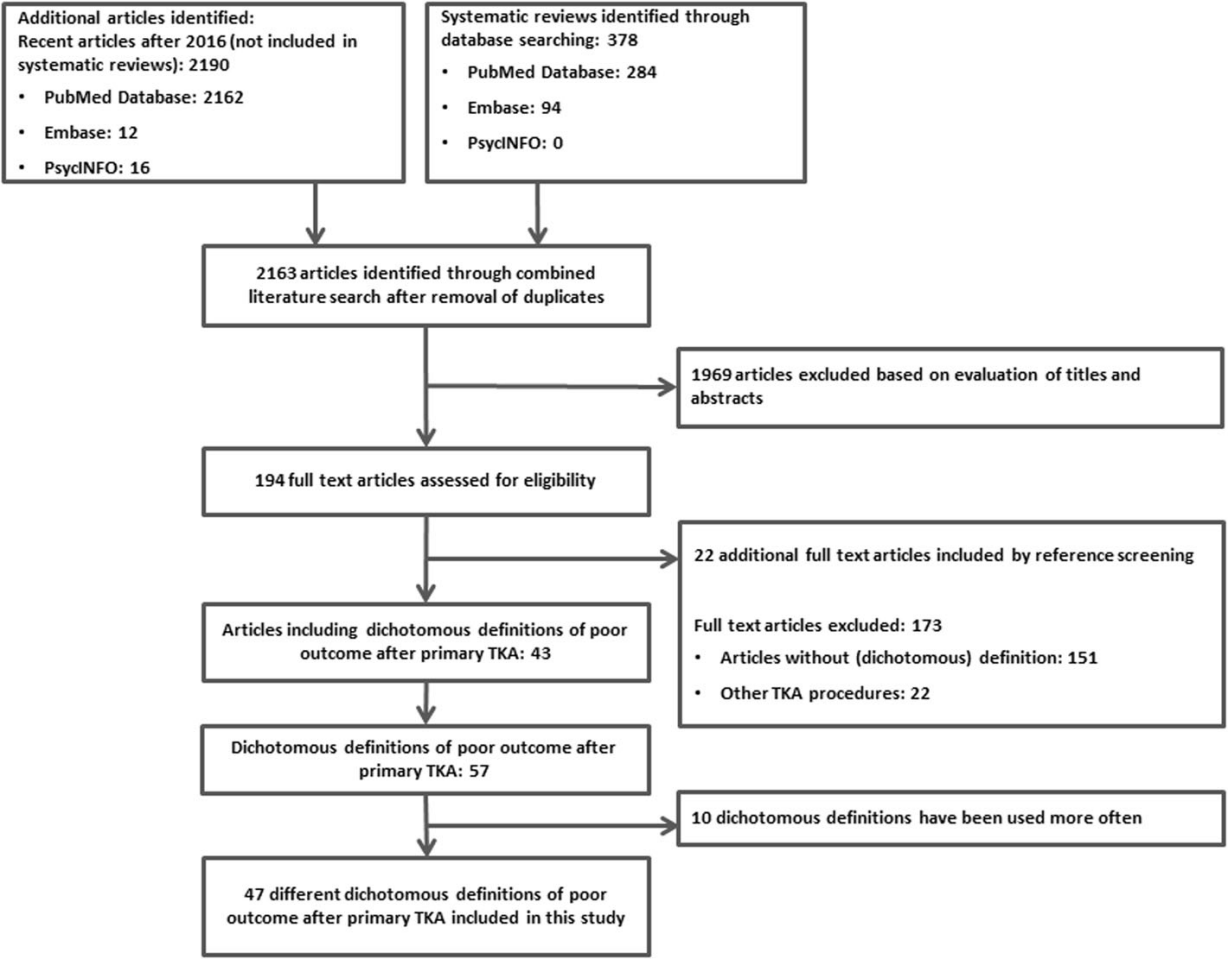
Flowchart of the study and definition selection

Previously used definitions for poor outcome after TKA in the literature, from 2000 up to October 2019 were identified and reviewed. We followed the following strategy to systematically identify definitions of poor outcome:

- An electronic search strategy was performed to retrieve systematic reviews (SRs) until 2016 in the PubMed Database, EMBASE and PsycINFO, on the outcome of TKA using search terms as “knee, arthroplasty, replacement, prosthesis, outcome measures, score and failure”. A detailed search strategy can be found in Fig. 1 and an example of the search strings is available (Additional file 1).
- To include more recent publications, articles from 2016 or later, were searched for definitions of poor outcome of TKA using the same search terms as for the SRs.
- Duplicates were excluded and search results were screened on title and abstract.
- All reference lists of included SRs were hand searched for relevant articles.
- Studies were assessed for eligibility by the in-and exclusion criteria.
- Subsequently, full texts screening of the eligible studies was carried out, for definitions of poor outcome after primary TKA.

### Study selection

Two reviewers (CvdE, MtM) independently selected eligible SRs and eligible recent publications from 2016 or later, describing a dichotomous definition for poor outcome after TKA in a certain domain or combination of domains. The reviewers (CvdE, MtM) independently selected eligible studies from the references lists of the, based on their titles, SRs and thereafter the eligibility criteria for studies.

### Eligibility criteria studies

Selected studies were assessed by the following eligibility criteria: 1) TKA patient population diagnosed with osteoarthritis; 2) primary TKA; 3) incorporating a dichotomous definition of poor outcome after primary TKA. 4) utilizing a controlled or prospective observational design (cohort and registry studies).

There were no restrictions on 1) sample size; 2) the type of primary implant; 3) follow-up moment of outcome; and 4) studies with missing data regarding primary- and secondary outcomes.

Studies with other TKA procedures (i.e. revision TKA, uni- or bicompartimental replacements) as study intervention, studies not written in the English language and articles without an abstract and/or access to the full text manuscript, were excluded.

### Selection of definitions

Full text evaluation of the eligible studies was carried out by one reviewer (MtM), to select eligible dichotomous definitions. When eligible studies used definitions from other articles, data on the definition was extracted from the original article. Criteria for definitions to be included in the review were: 1) included a threshold for dichotomizing; and 2) related to the concept of poor, worse or non-responder outcome as stated by the authors of that publication. Any uncertainty about the final selection of definitions and the extraction of data on the definitions was discussed with the second reviewer (CvdE) to achieve consensus.

### Data collection and synthesis

We extracted all dichotomous definitions of poor outcome after TKA using a data extraction form. Definitions were grouped according to outcome domains (e.g. pain, function, patient satisfaction), the following data were extracted:

- Study characteristics: author names, date of publication and length of study follow-up.
- Characteristics of the definition of poor outcome after primary TKA: number of domains, type of domains, outcome measure(s) used, type of threshold (absolute/relative cut-off value/change), value and time points).
- Additional background information on selected definitions of poor response provided by authors.

## Results

After removal of duplicates, a total of 329 SRs and 1834 articles after 2016 were screened on title and abstract (Fig. 1). The full texts of 48 SRs and 146 articles after 2016 were assessed for eligibility. In total 43 articles included 57 definitions of poor outcome after primary TKA. Forty-seven different dichotomous definitions of poor outcome after primary TKA were included in this study (Additional file 2).

There were 36 different unidimensional definitions, seven different definitions included two domains and four different definitions incorporated three domains. Eight different domains were identified in the 47 different definitions: pain, function, physical functioning, QoL, patient satisfaction, anxiety, depression, and patient global assessment. Pain (*N* = 28 different definitions), physical functioning (*N* = 17 different definitions) and patient satisfaction (*N* = 13 different definitions) domains were most frequently used in identified definitions of poor outcome after TKA. Patient satisfaction was only measured as unidimensional definitions with wide variation in wording of questions and answering categories. An absolute cut-off value of a certain outcome measure (*N* = 42 different definitions) was the most common type of threshold.

In the 47 different dichotomous definitions of poor outcome after primary TKA, we identified 14 different single item questions, two different self-composite question, one physician-assessed outcome measure, eight different PROMs and five different mixed outcome measures (combination of physician-assessed and patient reported). Single item questions measure one construct by asking for example the following question: ‘How satisfied are you with the outcome after TKA?’ Self-composite questions refer to composite questions of multiple items: ‘Overall limitations was defined as moderate/severe, if a patient had ≥ activities (walking, stairs, rising chair) with moderate or severe limitations (reference, <2 limitations)’. The Western Ontario and McMaster University Osteoarthritis Index (WOMAC) (*N* = 11 different dichotomous definitions) and the Visual Analogue Scale (VAS) (*N* = 5 different dichotomous definitions) were the most frequently used measurement instruments after the single item questions.

In 27 of the 57 selected definitions, additional background information was provided on the choice for the definitions and/or thresholds being used, other definitions were not substantiated (Additional file 3).

## Discussion

To our knowledge, this is the first inventory review that summarizes definitions of poor outcome after primary TKA presented in the literature. We found a total of 47 different definitions varying in nature and number of outcome domains involved, the type of response and the magnitude of change. A total of eight different dimensions were used in identified definitions of poor outcome: pain, function, physical functioning, health-related quality of life (HRQOL), patient satisfaction, anxiety, depression and patient global assessment. Patient satisfaction was used as single domain with a wide variation in wording of questions and answering categories. The absolute cut-off value was the most common type of threshold, with large variety in value and timing of follow-up. Our review stresses the need for an unambiguous, dichotomous definition for poor response after TKA to enable comparisons of the effectiveness of TKA among studies and among countries.

A remarkable finding of our review was that the majority of definitions used to describe poor outcome incorporate only one or two outcome domains. This finding does not seem to correspond with the conclusions by the OMERACT-OARSI initiative and the International Consortium for Health Outcomes Measurement (ICHOM). The OMERACT proposed a simplified set of responder criteria for (non-surgical) treatment of OA in clinical pharmaceutical trials. This set of responder criteria comprises relative and absolute changes in three domains: pain, physical functioning and patient global assessment [18, 19]. Also, the OMERACT TJR Working Group proposed a set of core domains (pain, function, patient satisfaction, revision, adverse events, and death) to evaluate joint replacement in randomized controlled trials [20]. Parallel, ICHOM has developed a set of patient-centered outcome measures and case-mix factors for evaluating, comparing and improving the treatment (both surgical and non-surgical) of patients with hip and knee OA, focusing on outcomes that matter to patients [21]. Pain, function, HRQOL and work status formed the core outcome domains, after a modified Delphi process [21]. Corresponding to this standard set and these responder criteria it seems important to measure poor response to TKA within multiple constructs to cover important key outcome domains to patients.

A great variety of thresholds is being used to measure poor response, ranging from an absolute cut-off point regarding patient dissatisfaction to composite measures incorporating relative changes or a MCID less than a certain value. Some studies used the inverse of the OMERACT-OARSI responder criteria “nonresponse” as a definition of poor outcome [22–26]. However, it is questionable whether “poor response” is the true opposite of “clinically meaningful response” as this definition implies that patients with smaller improvements will be part of the poor response group. The study by Mahler et al. showed a clear asymmetric magnitude of change, with a lower amount of change for patients who reported being worsened compared to the amount of improvement in patients who reported being improved [27]. In our opinion the amount of absolute or relative change in relevant constructs is therefore an important aspect of definitions of poor outcome.

In our opinion, strict, dichotomous definitions are necessary to interpret data on group level and to compare TKR outcome among hospitals, countries and over time. However, dichotomous data implies reduction of data and is therefore, less suitable for identifying factors underlying poor outcome. In particular, for individual patients, continuous outcomes are more suitable to monitor and evaluate specific health outcomes.

### Patient dissatisfaction

Patient satisfaction was used as single domain with a wide variation in wording of questions and answering categories, most frequently measured by single item questions (non-validated instruments) [14]. However, patient satisfaction is a multidimensional construct that may represent either satisfaction with outcome (e.g. knee function) of TKA or the process of care delivery, which all can be influenced by patients’ expectations [14, 28]. Halawi et al. explored subjective reasons for patient dissatisfaction after TJR and found different causes of patient dissatisfaction. The most common causes for dissatisfaction after TKA were persistent pain, functional limitation, surgical complication and reoperation, staff or quality of care issues and unmet expectations [28]. It is likely that different factors influence the construct of patient satisfaction, and therefore it is important to determine the different determinants that contribute to patient satisfaction after TKA according to the perspective of patients and orthopaedic surgeons.

### Different perspectives

The variety in definitions of poor outcome used could reflect different perspectives of physician, patient and clinical researcher. There are many studies reporting on the disagreement between the patient and physician in terms of their satisfaction with surgery [11, 29, 30]. It is conceivable that physicians tend to focus on aspects of their clinical evaluation (e.g. stability, range of motion and alignment), while patients are more likely to focus on the functionality of the knee during daily life activity. Moreover, the view of physicians and patients on the desired magnitude of improvement after TKA is not always concordant, as poor correlations were found between physician-assessed and PROMs [13]. Furthermore, most outcome measures have been developed according to the medical research perspective, which mainly address knee-specific measures like pain and function scores, and scarcely address mental functioning and consequences for social participation [31]. So far, the choice for definitions to describe response or non-response after TKA has been dominated by non-comprehensive physician-based scoring systems and PROMs in quantitative research but the perspectives of patients and orthopaedic surgeons regarding the definition of poor response have been relatively neglected.

### Additional background information

In 27 of the 57 selected definitions additional background information was provided to justify the choice for the definition and/or thresholds being used. Background information was extracted from the original publications. In particular, definitions of patient dissatisfaction were not substantiated and arbitrarily dichotomized.

This study has some limitations, as our searches for relevant articles were systematic but the data extraction was performed by a single reviewer. Although any uncertainty about the selection of definitions and the extraction of data on the definitions was discussed with the second reviewer (CvdE). This inventory review does provide a complete overview of definitions of poor response after TKA that could be of interest to a large group of physicians and researchers involved in defining outcomes after TKA. Furthermore, only studies published in English language were included. For this reason, it cannot be ruled out that some studies were not identified (language bias).

## Conclusions

In conclusion, this inventory review shows that many different heterogeneous definitions, incorporating several domains, for poor response to primary TKA are being used in the literature. Future research should focus on the perspectives and perceptions of orthopaedic surgeons and patients about constructs underlying poor response to TKA. Our findings stress the need for an consensus-based unambiguous, dichotomous definition of poor response to draw conclusions about the prevalence of poor-responders to TKA across hospitals and countries, and to identify patients at risk.

## Supplementary information

**Additional file 1.** Example search terms – for PubMed.

**Additional file 2.** Definitions of poor outcome after primary TKA.

**Additional file 3.** Additional background information about selected definitions.

## Data Availability

Data sharing is not applicable to this article as no datasets were generated or analysed for this manuscript.

## Abbreviations

OA: Osteoarthritis
TKA: Total knee arthroplasty
TJR: Total joint replacement
PROMS: Patient-reported outcome measure
QoL: Quality of life
HRQOL: Health-related quality of life
SRs: Systematic reviews
VAS: Visual analogue scale
WOMAC: Western Ontario and McMaster Universities Osteoarthritis Index
