# Supplementary material for: Definitions of poor outcome after total knee arthroplasty: an inventory review
Source: BMC Musculoskelet Disord. 2020 Jun 13;21:378. doi: 10.1186/s12891-020-03406-y (PMC7293790; doi:10.1186/s12891-020-03406-y)
Supplement: Supplementary file 1 — Additional file 1. Example search terms – for PubMed. [file 12891_2020_3406_MOESM1_ESM.pdf]

Supplementary file 1: Example search terms – for PubMed

PUBMED SRs:

(((((((("Arthroplasty, Knee"[Mesh] OR total knee arthroplasty [title]))) AND ("Outcome Assessment (Health Care)"[Mesh] OR Outcome [tiab] OR result [tiab] OR "Patient Reported Outcome Measures"[Mesh] OR patient reported [tiab] OR outcome score [tiab] OR PROM [tiab] OR clinical [tiab] OR function* [tiab] OR "Patient Satisfaction"[Mesh] OR satisfaction [tiab] OR "Prosthesis Failure"[Mesh] OR failure [tiab] OR "Pain"[Mesh] OR pain [tiab] OR "Postoperative Complications"[Mesh] OR complication* [tiab] OR "Postoperative Period"[Mesh] OR post-operative [tiab] OR "Long Term Adverse Effects"[Mesh] OR long-term [tiab]))) AND systematic [sb])

Filter used: Text availability: Full text

PUBMED articles after 2016:

((((((((("Arthroplasty, Knee"[Mesh] OR total knee arthroplasty [title]))) AND ("Outcome Assessment (Health Care)"[Mesh] OR Outcome [tiab] OR result [tiab] OR "Patient Reported Outcome Measures"[Mesh] OR patient reported [tiab] OR outcome score [tiab] OR PROM [tiab] OR clinical [tiab] OR function* [tiab] OR "Patient Satisfaction"[Mesh] OR satisfaction [tiab] OR "Prosthesis Failure"[Mesh] OR failure [tiab] OR "Pain"[Mesh] OR pain [tiab] OR "Postoperative Complications"[Mesh] OR complication* [tiab] OR "Postoperative Period"[Mesh] OR post-operative [tiab] OR "Long Term Adverse Effects"[Mesh] OR long-term [tiab]))))

Filters used: Publication dates (From 2016/01/01 to 2019/05/14); Text availability: Full text
