# Supplementary material for: Definitions of poor outcome after total knee arthroplasty: an inventory review
Source: BMC Musculoskelet Disord. 2020 Jun 13;21:378. doi: 10.1186/s12891-020-03406-y (PMC7293790; doi:10.1186/s12891-020-03406-y)
Supplement: Supplementary file 2 — Additional file 2. Definitions of poor outcome after primary TKA. [file 12891_2020_3406_MOESM2_ESM.doc]

| **Definitions of poor outcome after primary TKA** | | | | |
| --- | --- | --- | --- | --- |
| **Domain** | **Outcome measures used** | **Absolute/relative**  **cut-off value/ change** | **Value** | **Time points used** |
| **Unidimensional (single domain used)** | | | | |
| **Pain** | VAS pain scale (0-5)  VAS pain scale (0-100) | *absolute cut-off* | - >0 *(Forsythe et al., 2008* (1)*)* - >40 *(Brander et al., 2003* (2)*; Brander et al., 2007* (3)*)* | 24M  1, 3, 6, 12M |
|  | VAS pain at rest scale (0-100) | *absolute cut-off* | ≥1 *(Lundblad et al., 2008* (4)*)* | 18M |
|  | VAS pain with movement scale (0-100) | *absolute cut-off* | ≥1 *(Lundblad et al., 2008* (4)*)* | 18M |
|  | WOMAC pain subscale | *relative change* | <50% improvement from baseline to 6M FU *(Riddle et al., 2010* (5)*)* | 6M |
|  | WOMAC pain subscale | *absolute change* | MCID ≤4 *(Riddle et al., 2010* (5)*)* | 6M |
|  | WOMAC pain subscale (0-100) | *absolute cut-off* | <100 and present for at least the past 3M *(Wylde et al., 2011* (6)*)* | 3M |
|  | WOMAC pain subscale | *absolute cut-off* | Moderate or worse response in any of the five questions of the pain scale *(Czurda et al., 2010* (7)*)* | at least 18M |
|  | KSS pain subscale | *absolute cut-off* | ≤30 (*Fisher et al., 2007* (8)*)* | 12M |
|  | IKSS pain subscale (0-50) | *absolute cut-off* | <30 *(Dowsey et al., 2012* (9)*)* | 12, 24M |
|  | McGill Pain Index (0-5) | *absolute cut-off* | >0 *(Forsythe et al., 2008* (1)*)* | 24M |
|  | BPI pain scale (0-10) | *absolute cut-off* | ≥3 *(Masselin- Dubois et al., 2013* (10)*)* | 3M |
|  | AKSS pain subscale (0-7) | *absolute cut-off* | ≥5 *(Elson et al., 2006* (11)*)* | 5Y |
|  | NRS pain scale (0-10) | *absolute cut-off* | >3 *(Pinto et al., 2013* (12)*)* | 4 to 6M |
|  | Single item question pain | *absolute cut-off* | Category: moderate and severe pain *(Singh et al., 2010/06* (13)*; Singh et al., 2014* (14)*)* | 2, 5Y |
| **Function (impairment)** | Knee flexion function | *absolute cut-off* | <90° *(Fisher et al., 2007* (8)*; Boonen et al., 2016* (15)*)* | 1, 2Y |
|  | Single item question function | *absolute cut-off* | Categories: ‘somewhat better, same, worse’ *(Singh et al., 2010/06* (13)*)* | 2, 5Y |
| **Physical Functioning** | IKSS functioning subscale (0-100) | *absolute cut-off* | <60 *(Dowsey et al., 2012* (9)*)* | 12, 24M |
|  | WOMAC functioning subscale | *relative change* | <50% improvement from baseline to 6M FU *(Riddle et al., 2010* (5)*)* | 6M |
|  | WOMAC functioning  subscale | *absolute change* | MCID ≤15 *(Riddle et al., 2010* (5)*)* | 6M |
|  | Single item question functioning | *absolute cut-off* | Maximum walk time ≤15 min *(Yong-Hao Pua et al., 2016* (16)*)* | 6M |
|  | Self-composite question functioning | *absolute cut-off* | ≥2 activities (walking, stairs, rising chair) with moderate-severe limitations *(Singh et al., 2010/04* (17)*; Singh et al., 2010/06* (13)*; Singh et al., 2014* (14)*)* | 2, 5Y |
|  | Self-composite question functioning | *absolute cut-off* | No *(Filbay et al.,2019* (18)*)* | 12M |
| **Satisfaction** | VAS satisfaction scale (0-100) | *absolute cut-off* | ≥50 *(Judge et al., 2012* (19)*)* | 6M |
|  | New KSS satisfaction subscale (0-40) | *absolute cut-off* | <20 *(Onsem et al., 2016* (20)*)* | 3M |
|  | Single item question satisfaction | *absolute cut-off* | - Dissatisfied *(Aggarwal et al., 2013* (21)*)* - Dissatisfied (=somewhat dissatisfied and very dissatisfied) *(Gandhi et al., 2009* (22)*; Filbay et al., 2019* (18)*)* - Dissatisfied (=unsure and dissatisfied) *(Scott et al., 2010* (23)*)* - Unsatisfied (=unsatisfied and very unsatisfied) *(Bierke et al., 2017* (24)*)* - Less satisfied (=moderately satisfied, neutral, moderately dissatisfied and very dissatisfied) *(Vissers et al., 2010* (25)*)* - Unsatisfied (=somewhat dissatisfied and dissatisfied) *(Escobar et al., 2011* (26)*)* - Dissatisfied (=uncertain and dissatisfied) *(Scott et al., 2016* (23)*)* - Dissatisfied (=no and I’m not sure) *(Jacobs et al., 2014* (27)*)* - Dissatisfied (=no) *(Kunze et al., 2018* (28)*)* - Not Satisfied *(Halawi et al., 2019* (29)*)* | 12M  12M  12M  6M,  12M  6M  12M  12M  2 to 5 Y  12M  12M |
|  | Single item question satisfaction | *relative cut-off* | ≤ 50% *(Merle-Vincent et al., 2011* (30)*)* | 24M |
| **Multidimensional (two domains used)** | | | | |
| **Anxiety + Depression** | HADS anxiety & depression subscales (0-100)/ (partial scale: 0-18 & total scale: 0-36) | *absolute cut-off* | - ≥8 *(Vissers et al., 2010* (25)*)* - Equivalent or higher scores than 5 for partial scores and 10 for the total score *(Caracciolo et al., 2005* (31)*)* | 6M  Discharge |
| **Pain + Physical functioning** | OKS pain & functioning subscales (0-48) | *absolute cut-off* | <27 *(Seah et al., 2017* (32)*)* | 6M |
|  | OKS pain & functioning subscales | *absolute chang*e | - MCID ≤5 *(Alzahrani et al., 2011* (33)*)* - MCID ≤6 *(Filbay et al.,2019* (18)*)* | 12M |
|  | WOMAC pain & functioning subscales (0-100) | *absolute cut-off* | <60 *(Katz et al., 2007* (34)*)* | 2Y |
|  | WOMAC pain & functioning subscales | *relative change* | <10% difference between the mean pre- and post-operative scores of the pain and function dimensions *(Nunez et al., 2007* (35)*; Nunez et al., 2009* (36)*)* | 36M, 7Y |
| **Multidimensional (three domains used)** | | | | |
| **Pain + Function (impairment) + Global assessment** | OMERACT-OARSI responder criteria (WOMAC pain & functioning subscales & global score) | *absolute & relative change* | Non-responder: <50% improvement and less than an absolute improvement of 20 points in either pain or function OR  if there was improvement in 2 of the 3 following: pain of <20% and an absolute change of <10, function <20% and an absolute change of <10*, global improvement of* <20% and an absolute change of <10 *(Dowsey et al., 2016* (37)*; Riddle et al., 2017* (38)*; Dowsey et al., 2017* (39)*; Weber et al., 2018* (40)*)* | 12M |
| **Pain + Function (impairment) + Physical functioning** | WOMAC pain, stiffness & functioning subscales (0-100) | *absolute cut-off* | >40.4 *(Lungu et al., 2014* (41)*)* | 6M |
|  | WOMAC pain, stiffness & functioning subscales | *absolute change* | MCID <7.5 *(Alzahrani et al., 2011* (33)*)* | 12M |
| **Function (impairment) + Physical functioning + HR-QoL** | KSS knee & function subscales & UCLA functioning scale & SF-12 physical & mental subscales | *absolute change* | Deterioration of ≥1 compared to preoperative *(Kokubun et al., 2017* (42)*)* | 6W, 12W, 6M |
| *AKSS American Knee Society Score, BPI Brief Pain Inventory, HADS Hospital Anxiety and Depression Scale, HR-QoL* health related quality of life*, IKSS International Knee Society Score, KSS Knee Society Score, New KSS New Knee Society Score, NRS Numerical Rating Scale, OKS Oxford Knee Score, OMERACT-OARSI Outcome Measures in Rheumatology committee and Osteoarthritis Research Society International committee, PRI Pain Rating Index, SF-12 12 Item Short Form Survey, UCLA University of California-Los Angeles, VAS Visual Analogue Scale, WOMAC Western Ontario and McMaster Universities Osteoarthritis Index*  *Time points: W weeks, M months, Y years* | | | | |

1. Forsythe M, Dunbar M, Hennigar A, Sullivan M. Prospective relation between catastrophizing and residual pain. Pain Res Manag. 2008;13(4):335–41.

2. Brander V, Stulberg S, Adams A, Harden R, Bruehl S, Stanos S, et al. Predicting Total Knee Replacement Pain. Clin Orthop Relat Res. 2003;(416):27–36.

3. Brander V, Gondek S, Martin E, Stulberg S. Pain and depression influence outcome 5 years after knee replacement surgery. Clin Orthop Relat Res. 2007;(464):21–6.

4. Lundblad H, Kreicbergs A, Jansson K. Prediction of persistent pain after total knee replacement for osteoarthritis. J Bone Joint Surg Br. 2008;90-B(2):166–71.

5. Riddle D, Wade J, Jiranek W, Kong X. Preoperative pain catastrophizing predicts pain outcome after knee arthroplasty. Clin Orthop Relat Res. 2010;468(3):798–806.

6. Wylde V, Hewlett S, Learmonth I, Dieppe P. Persistent pain after joint replacement: Prevalence, sensory qualities, and postoperative determinants. Pain. 2011;152(3):566–72.

7. Czurda T, Fennema P, Baumgartner M, Ritschl P. The association between component malalignment and post-operative pain following navigation-assisted total knee arthroplasty: results of a cohort/nested case-control study. Knee Surg Sport Traumatol Arthrosc. 2010;18(7):863–9.

8. Fisher D, Dierckman B, Watts M, Davis K. Looks Good But Feels Bad: Factors That Contribute to Poor Results After Total Knee Arthroplasty. J Arthroplasty. 2007;22(6):39–42.

9. Dowsey M, Nikpour M, Dieppe P, Choong P. Associations between pre-operative radiographic changes and outcomes after total knee joint replacement for osteoarthritis. Osteoarthr Cartil. 2012;20(10):1095–102.

10. Masselin-Dubois A, Attal N, Fletcher D, Jayr C, Albi A, Fermanian J, et al. Are psychological predictors of chronic postsurgical pain dependent on the surgical model? A comparison of total knee arthroplasty and breast surgery for cancer. J Pain. 2013;14(8):854–64.

11. Elson D, Brenkel I. Predicting Pain After Total Knee Arthroplasty. J Arthroplasty. 2006;21(7):1047–53.

12. Pinto P, McIntyre T, Ferrero R, Almeida A, Araújo-Soares V. Risk Factors for Moderate and Severe Persistent Pain in Patients Undergoing Total Knee and Hip Arthroplasty: A Prospective Predictive Study. PLoS One. 2013;8(9):1–11.

13. Singh J, O’Byrne M, Colligan R, Lewallen D. Pessimistic explanatory style: a psychological risk factor for poor pain and functional outcomes two years after knee replacement. J Bone Joint Surg Br. 2010;92-B(6):799–806.

14. Singh J, Lewallen D. Are outcomes after total knee arthroplasty worsening over time? A time-trends study of activity limitation and pain outcomes. BMC Musculoskelet Disord. 2014;15(1):1–9.

15. Boonen B, Schotanus M, Kerens B, van der Weegen W, Hoekstra H, Kort N. No difference in clinical outcome between patient-matched positioning guides and conventional instrumented total knee arthroplasty two years post-operatively. Bone Jt J. 2016;98B(7):939–44.

16. Pua YH, Seah F, Clark R, Poon C, Tan J, Chong H. Development of a prediction model to estimate the risk of walking limitations in patients with total knee arthroplasty. J Rheumatol. 2016;43(2):419–26.

17. Singh J, O’Byrne M, Harmsen S, Lewallen D. Predictors of moderate-severe functional limitations after primary total arthroplasty (TKA): 4,701 TKAs at 2-years and 2,935 at 5-years. Osteoarthr Cartil. 2010;18(4):2–15.

18. Filbay S, Hons B. Evaluating patients’ expectations from a novel patient-centered perspective predicts knee arthroplasty outcome. J Arthroplasty. 2019;33(7):2146–52.

19. Judge A, Arden N, Cooper C, Kassim javaid M, Carr A, Field R, et al. Predictors of outcomes of total knee replacement surgery. Rheumatology. 2012;51(10):1804–13.

20. van Onsem S, van der Straeten C, Arnout N, Deprez P, van Damme G, Victor J. A new prediction model for patient satisfaction after total knee arthroplasty. J Arthroplasty. 2016;31(12):2660–7.

21. Aggarwal A, Agrawal A. Mobile vs fixed-bearing total knee arthroplasty performed by a single surgeon. A 4- to 6.5-year randomized, prospective, controlled, double-blinded study. J Arthroplasty. 2013;28(10):1712–6.

22. Gandhi R, Davey J, Mahomed N. Patient Expectations Predict Greater Pain Relief with Joint Arthroplasty. J Arthroplasty. 2009;24(5):716–21.

23. Scott CEH, Howie CR, Macdonald D, Biant LC. Predicting dissatisfaction following total knee replacement: a prospective study of 1217 patients. J Bone Jt Surg Br. 2010;92(9):1253–8.

24. Bierke S, Petersen W. Influence of anxiety and pain catastrophizing on the course of pain within the first year after uncomplicated total knee replacement: a prospective study. Arch Orthop Trauma Surg. 2017;137(12):1735–42.

25. Vissers M, de Groot I, Reijman M, Bussmann J, Stam H, Verhaar J. Functional capacity and actual daily activity do not contribute to patient satisfaction after total knee arthroplasty. BMC Musculoskelet Disord. 2010;11(121):1–8.

26. Escobar A, Gonzalez M, Quintana J, Vrotsou K, Bilbao A, Herrera-Espiñeira C, et al. Patient acceptable symptom state and OMERACT-OARSI set of responder criteria in joint replacement. Identification of cut-off values. Osteoarthr Cartil. 2012;20(2):87–92.

27. Jacobs C, Christensen C, Karthikeyan T. Patient and Intraoperative Factors Influencing Satisfaction Two to Five Years After Primary Total Knee Arthroplasty. J Arthroplasty. 2014;29(8):1576–9.

28. Kunze K, Akram F, Fuller B, Zabawa L, Sporer S, Levine B. Internal Validation of a Predictive Model for Satisfaction After Primary Total Knee Arthroplasty. J Arthroplasty. 2019;34(4):663–70.

29. Halawi M, Jongbloed W, Baron S, Savoy L, Williams V, Cote M. Patient dissatisfaction after primary total joint arthroplasty: the patient perspective. J Arthroplasty. 2019;34(6):1093–6.

30. Merle-Vincent F, Couris C, Schott A, Conrozier T, Piperno M, Mathieu P, et al. Factors predicting patient satisfaction 2 years after total knee arthroplasty for osteoarthritis. Jt Bone Spine. 2011;78(4):383–6.

31. Caracciolo B, Giaquinto S. Self-perceived distress and self-perceived functional recovery after recent total hip and knee arthroplasty. Arch Gerontol Geriatr. 2005;41(2):177–81.

32. Seah R, Lim W, Lo N, Yew A, Chong H, Yeo S. Unexplained Pain Post Total Knee Arthroplasty With an Oxford Knee Score ≥20 at 6 Months Predicts Good 2-Year Outcome. J Arthroplasty. 2017;32(3):807–10.

33. Alzahrani K, Gandhi R, DeBeer J, Petruccelli D, Mahomed N. Prevalence of clinically significant improvement following total knee replacement. J Rheumatol. 2011;38(4):753–9.

34. Katz J, Mahomed N, Baron J, Barrett J, Fossel A, Creel A, et al. Association of hospital and surgeon procedure volume with patient-centered outcomes of total knee replacement in a population-based cohort of patients age 65 years and older. Arthritis Rheum. 2007;56(2):568–74.

35. Núñez M, Núñez E, Luis del Val J, Ortega R, Segur J, Hernández M, et al. Health-related quality of life in patients with osteoarthritis after total knee replacement: Factors influencing outcomes at 36 months of follow-up. Osteoarthr Cartil. 2007;15(9):1001–7.

36. Núñez M, Lozano L, Núñez E, Segur J, Sastre S, Maculé F, et al. Total knee replacement and health-related quality of life: Factors influencing long-term outcomes. Arthritis Care Res. 2009;61(8):1062–9.

37. Dowsey M, Spelman T, Choong P. Development of a Prognostic Nomogram for Predicting the Probability of Nonresponse to Total Knee Arthroplasty 1 Year After Surgery. J Arthroplasty. 2016;31(8):1654–60.

38. Riddle D, Golladay G, Jiranek W, Perera R. External validation of a prognostic model for predicting nonresponse following knee arthroplasty. J Arthroplasty. 2017;32(4):1153–8.

39. Dowsey M, Robertsson O, Sundberg M, Lohmander L, Choong P, W-Dahl A. Variations in pain and function before and after total knee arthroplasty: a comparison between Swedish and Australian cohorts. Osteoarthr Cartil. 2017;25(6):885–91.

40. Weber M, Craiovan B, Woerner M, Schwarz T, Grifka J, Renkawitz T. Predictors of outcome after primary total joint replacement. J Arthroplasty. 2018;33(2):431–5.

41. Lungu E, Desmeules F, Dionne C, Belzile É, Vendittoli P. Prediction of poor outcomes six months following total knee arthroplasty in patients awaiting surgery. BMC Musculoskelet Disord. 2014;15(1):1–11.

42. Kokubun B, Manista G, Courtney P, Kearns S, Levine B. Intra-Articular Knee Injections Before Total Knee Arthroplasty: Outcomes and Complication Rates. J Arthroplasty. 2017;32(6):1798–802.
