# Supplementary material for: Definitions of poor outcome after total knee arthroplasty: an inventory review
Source: BMC Musculoskelet Disord. 2020 Jun 13;21:378. doi: 10.1186/s12891-020-03406-y (PMC7293790; doi:10.1186/s12891-020-03406-y)
Supplement: Supplementary file 3 — Additional file 3. Additional background information about selected definitions. [file 12891_2020_3406_MOESM3_ESM.docx]

| **Additional background information about selected definitions of poor response provided by authors** | |
| --- | --- |
| **Definition** | **Additional information** |
| VAS pain >0  (Forsythe, 2008 (33)) | “Sample was dichotomized into definitive ‘pain’(>0) and ‘no pain (0)’ groups, rather than arbitrarily dichotomizing the population as having mild or moderate pain.” |
| VAS pain >40  (Brander, 2007 (35)) | “Cut-offs were defined according to Dolan and Sutton(1).” |
| VAS pain at rest ≥1  (Lundblad, 2008 (36)) | “Dichotomisation was performed at the median value.” |
| VAS pain with movement ≥1  (Lundblad, 2008 (36)) | “Dichotomisation was performed at the median value.” |
| WOMAC pain <50%  (Riddle, 2010 (32)) | “Patients were dichotomized into those who did and those who did not improve by 50% or greater based on changes in WOMAC pain scores from baseline to 6 months postsurgery. Our rationale for this approach is that large sample studies generally indicate average improvement for patients is approximately 50% relative to initial scores.” |
| WOMAC pain MCID ≤4  (Riddle, 2010 (32)) | “For 6-month change scores, we dichotomized our sample based on whether changes exceed the 6-month minimal clinically important difference (MCID)(2). These changes were greater than 4 points for WOMAC pain.” |
| IKSS pain <30  (Dowsey et al, 2012 (40)) | “Pain was dichotomised into two categories based on severity at 12 months and 2 years (those with IKSS pain score ≥30) were classified as having none to mild pain and those with IKSS pain score <30 were classified as having moderate to severe pain(3).” |
| McGill Pain Index >0  (Forsythe et al, 2008 (33)) | “Sample was dichotomized into definitive ‘pain’ and ‘no pain’ groups, rather than arbitrarily dichotomizing the population as having mild or moderate pain.” |
| Brief Pain Inventory ≥3  (Masselin- Dubois, 2013 (41)) | “Clinically meaningful pain was considered to be present if patients rated their average pain or pain right now as ≥3/10 on the BPI. This cutoff corresponds to at least moderate pain with a potential impact on physical or emotional functioning.” |
| NRS pain >3  (Pinto, 2013 (43)) | “Patients reporting significant ‘‘worst pain’’ levels in the surgical area (NRS >3) were considered as being PPSP positive, similar to previous studies (4). ………. this cut-off was based on previous recommendations considering the differential impact of pain levels above 3 (5,6).” |
| Single item question function: somewhat better, same, worse  (Singh, 2010/06 (44)) | “Patients in the category of ‘much better’ were compared with those in the reference category comprising ‘somewhat better, same, worse’, Based on the fact that, typically, TKR is an extremely successful procedure and most patients aim to achieve much better knee function than pre-operatively.” |
| IKSS functioning <60  (Dowsey, 2012 (40)) | According to Asif et al. (7). |
| WOMAC functioning <50% improvement from baseline to 6M FU  (Riddle, 2010 (32)) | “Patients were dichotomized into those who did and those who did not improve by 50% or greater based on changes in WOMAC function scores from baseline to 6 months postsurgery. Our rationale for this approach is that large sample studies generally indicate average improvement for patients is approximately 50% relative to initial scores.” |
| WOMAC functioning MCID ≤15  (Riddle, 2010 (32)) | “For 6-month change scores, we dichotomized our sample based on whether changes exceed the 6-month minimal clinically important difference (MCID)(2). These changes were greater than 15 points for WOMAC function.” |
| New KSS, satisfaction <20  (Onsem, 2016 (51)) | “A score of more than 20 meant that the patient selected “satisfied” at least one time and a score lower than 20 that the patient selected “dissatisfied” at least once. We considered 20 as the cut off value for satisfaction.” |
| HADS anxiety & depression ≥8  (Vissers, 2010 (55)) | According to Zigmond et al. (8). |
| HADS Equivalent or higher scores than 5 for partial scores and 10 for the total score  (Caracciolo, 2005 (60)) | “The cut-off scores for the Italian version are fixed at 5 for the partial scores and at 10 for the total score. Equivalent or higher scores classify subjects as psychologically distressed. The evaluations were established at admission and at discharge.” |
| OKS pain & functioning <27  (Seah, 2017 (61)) | “Unexplained pain after primary TKA and an Oxford knee score (OKS) less than 27 (which is considered a poor outcome (9) at 6 months.” |
| OKS pain & functioning MCID≤5  (Alzahrani, 2011 (62)) | “We defined the MCID for the OKS as a 5-point change or less, as suggested by Murray et al. (10).” |
| OKS pain & functioning MCID≤6  (Filbay, 2019 (49)) | “The MIC for the OKS following TKA at an individual level, has been estimated to be 7-points (considered as the minimal amount of change necessary to distinguish between patients ‘a little better’ from those ‘about the same’ in a UK sample of 94,502 individuals undergoing knee arthroplasty) (11). To enhance interpretation of findings and maintain consistency with other binary outcomes, the OKS was dichotomised using a cut-off of 7-points, whereby patients reporting an improvement of 6-points or less on the OKS between baseline and one-year follow-up were categorised as ‘not achieving OKS MIC.” |
| WOMAC pain & functioning <60  (Katz, 2007 (63)) | “Scores <60 indicated poor outcome. Sixty is a typical preoperative score (12).” |
| OMERACT-OARSI responder criteria (WOMAC, pain & functioning and global score): Non-responder: <50% improvement and less than an absolute improvement of 20 points in either pain or function OR  if there was improvement in 2 of the 3 following: pain of <20% and an absolute change of <10, function <20% and an absolute change of <10*, global improvement of* <20% and an absolute change of <10  (Dowsey, 2016 (66) & 2017 (24); Riddle, 2017 (22); Weber, 2018 (26)) | References made to Pham et al. (13). |
| WOMAC pain, stiffness & functioning >40.4  (Lungu, 2014 (67)) | “As there is no universal agreement on what is considered poor outcome following TKA surgery, it was defined as the last quintile of the six-month postoperative WOMAC score (i.e. WOMAC score > 40.4); a satisfactory outcome was defined….. distribution (i.e. score ≤ 40.4).” |
| WOMAC pain, stiffness & functioning MCID<7.5  (Alzahrani, 2011 (62)) | According to Mahomed et al. (14). |

1. Dolan P, Sutton M. Mapping visual analogue scale health state valuations onto standard gamble and time trade-off values. Soc Sci Med. 1997;44(10):1519–30.

2. Escobar A, Quintana J, Bilbao A, Aróstegui I, Lafuente I, Vidaurreta I. Responsiveness and clinically important differences for the WOMAC and SF-36 after total knee replacement. Osteoarthr Cartil. 2007;15(3):273–80.

3. Insall JN, Dorr LD, Scott RD, Scott WN. Rationale of the Knee Society clinical rating system. Clin Orthop Relat Res. 1989;248(November (248)):13–4.

4. Vuorenmaa M, Ylinen J, Kiviranta I, Intke A, Kautiainen H, Mälkiä E, et al. Changes in pain and physical function during waiting time and 3 months after knee joint arthroplasty. J Rehabil Med. 2008;40(7):570–5.

5. Dihle A, Helseth S, Paul S, Miaskowski C. The exploration of the establishment of cutpoints to categorize the severity of acute postoperative pain. Clin J Pain. 2006;22(7):617–24.

6. Bodian C, Freedman G, Hossain S, Eisenkraft J, Beilin Y. The visual analog scale for pain. Clinical significance in postoperative patients. Anesthesiology. 2001;95(6):1356–61.

7. Asif S, Choon D. Midterm results of cemented Press Fit Condylar Sigma total knee arthroplasty system. J Orthop Surg (Hong Kong). 2005;13(3):280–4.

8. Zigmond A, Snaith R. The Hospital Anxiety and Depression Scale. Acta psychiatr scand. 1983;67:361–70.

9. Pynsent P, Adams D, Disney S. The Oxford hip and knee outcome questionnaires for arthroplasty. J Bone Jt Surg Br. 2005;87-B(2):241–8.

10. Murray D, Fitzpatrick R, Rogers K, Pandit H, Beard D, Carr A, et al. The use of the Oxford hip and knee scores. J Bone Jt Surg Br. 2007;89-B(8):1010–4.

11. Beard D, Harris K, Dawson J, Doll H, Murray D, Carr A, et al. Meaningful changes for the Oxford hip and knee scores after joint replacement surgery. J Clin Epidemiol. 2015;68(1):73–9.

12. Fortin P, Clarke A, Joseph L, Liang M, Tanzer M, Ferland D, et al. Outcomes of total hip and knee replacement: Preoperative functional status predicts outcomes at six months after surgery. Arthritis Rheum. 1999;42(8):1722–8.

13. Pham T, Van Der Heijde D, Lassere M, Altman R, Anderson J, Bellamy N, et al. Outcome variables for osteoarthritis clinical trials: The OMERACT-OARSI set of responder criteria. J Rheumatol. 2003;30(7):1648–54.

14. Mahomed N, Davis A, Hawker G, Badley E, Davey J, Syed K, et al. Inpatient compared with home-based rehabilitation following primary unilateral total hip or knee replacement: A randomized controlled trial. J Bone Jt Surg Am. 2008;90(8):1673–80.
